# Non-pharmacological Interventions for Muscle Cramps and Pain in Patients With Cirrhosis: A Systematic Review

**DOI:** 10.7759/cureus.64859

**Published:** 2024-07-18

**Authors:** Ryan Muller, Jonathan Dranoff, Alyssa A Grimshaw, Lori Bastian, Craig Gunderson

**Affiliations:** 1 Physical Medicine and Rehabilitation, Pain Research, Informatics, Multi-morbidities, and Education (PRIME) Center, Veterans Affairs (VA) Connecticut Healthcare System, West Haven, USA; 2 Gastroenterology, Veterans Affairs (VA) Connecticut Healthcare System, West Haven, USA; 3 Library Science, Harvey Cushing/John Hay Whitney Medical Library, Yale University, New Haven, USA; 4 Internal Medicine, Pain Research, Informatics, Multi-morbidities, and Education (PRIME) Center, Veterans Affairs (VA) Connecticut Healthcare System, West Haven, USA; 5 Hospital-Based Medicine, Hospital Operations, Veterans Affairs (VA) Connecticut Healthcare System, West Haven, USA

**Keywords:** : pain, a systematic review, non-pharmacological treatment, nutritional intervention, cirrhosis of the liver

## Abstract

Despite the high prevalence of pain and challenges associated with traditional pharmacological pain management in patients with cirrhosis, little is known about the safety and effectiveness of non-pharmacological management of pain in this patient population. A systematic literature search of published studies was conducted in nine databases from inception through January 11, 2023, including any clinical trial, cohort, or case-control study of non-pharmacological pain interventions in adult patients with cirrhosis. Studies using nutritional supplements were included. The primary and secondary outcomes for this review were pain/analgesic effect and safety, respectively. Two reviewers independently performed data extraction and risk of bias assessment. Of the 4,087 studies initially screened, 11 studies representing 340 patients ultimately met inclusion criteria, including seven observational and four randomized controlled trials. Five studies reported muscle cramp severity, four reported muscle cramp frequency, and two reported non-cramp pain. Oral zinc sulfate, L-carnitine, and taurine were reported to decrease cramp frequency. Oral vitamin E, oral zinc sulfate, L-carnitine, taurine, and pickle juice decreased cramp severity. Curcumin supplementation, resistance training, and stretching and walking programs improved non-cramp pain. Mild adverse events were reported in four studies. The risk of bias was moderate to high for all studies, largely due to missing data, study design, and a lack of blinding of participants. Numerous nutritional and non-pharmacological interventions have been reported to be safe and effective for the treatment of pain and painful muscle cramps in patients with cirrhosis. However, further research is needed to better determine the efficacy, safety, and optimal frequency and dosage of interventions.

## Introduction and background

Cirrhosis is a growing public health concern in the US [1]. According to provisional data from the National Vital Statistics System, chronic liver disease and cirrhosis ranked ninth as underlying causes of death in the US [2]. Many patients with cirrhosis experience pain and/or painful muscle cramps, with approximately 80% of patients reporting pain and 75% reporting pain-related disability [3,4]. Furthermore, chronic pain is experienced by over half of patients with cirrhosis [3]. Fibromyalgia-like symptoms, depression, opioid use, sleep disturbance, and depression are also more prevalent in patients with liver disease [3,4].

The treatment of pain in cirrhosis patients is complicated by the absolute and relative contraindications to pharmacological therapies that must be considered in this patient population. Many analgesic treatment options, such as nonsteroidal anti-inflammatory drugs and paracetamol, are associated with drug-induced liver injury. Patients with chronic liver disease also have an increased risk of adverse drug reactions such as constipation, renal dysfunction, and oversedation as a function of altered drug metabolism in this population [1,4]. Thus, pain control in patients with cirrhosis is often inadequately addressed. Despite the prevalence of pain and complications associated with pharmacological management in patients with cirrhosis, little is known about the effectiveness and safety of the management of painful symptoms with non-pharmacological interventions in this population. An in-depth investigation of the non-pharmacological management of patients with cirrhosis is needed to address this treatment deficit. Therefore, the objective of our study was to conduct a systematic review summarizing the published literature examining the effectiveness and safety of non-pharmacological interventions for pain in patients with cirrhosis.

## Review

Materials and methods

The protocol for this study was pre-registered at PROSPERO (ID: CRD42023401414). We reported this study consistent with the Meta-analysis of Observational Studies in Epidemiology (MOOSE) guidelines [5] and the Preferred Reporting Items for Systematic Reviews and Meta-Analyses (PRISMA) [6] statements (Appendices A, B, and C).

Data Sources and Search Strategy

An exhaustive search of the literature was conducted by a medical research librarian (AG) in Ovid AMED, CINAHL, Cochrane Library, Google Scholar, Ovid Embase, Ovid MEDLINE, PubMed, Scopus, and Web of Science Core Collection databases to find relevant articles published from the inception of each database to January 13, 2023. Databases were searched using a combination of keywords and controlled vocabulary for cirrhosis and pain management or pain and pain management interventions. The search was conducted without restrictions on language, publication type, or publication year and was peer-reviewed by a second medical librarian, according to the Peer Review of Electronic Search Strategies (PRESS) [7]. In order to identify additional relevant studies that may not have been retrieved by the database search, forward and backward citation chasing was performed using citationchaser [8]. The detailed search strategy can be downloaded from Open Science Framework (https://osf.io/57tpc).

Study Selection

The Population, Intervention, Comparison, Outcomes and Study (PICO) [9] criteria were used to determine the eligibility of the articles based on the type of study design, type of population, type of exposure and outcome, number of participants, and follow-up period. Non-pharmacological interventions, including nutritional supplements, were the exposure. The primary outcome was pain/analgesic effect, and the secondary outcome was safety. Comparative study designs (randomized controlled trial (RCT), cross-sectional, cohort, and case-control) that assessed the effectiveness and/or safety of nutritional and/or non-pharmacological interventions in patients with cirrhosis in adult populations were included. Studies in which the participants were children were excluded. Studies presenting unoriginal data and containing minimal information in the methods and results sections were not included. Studies of patients who had undergone liver transplants or were on a transplant list and studies using prescription medications as an intervention were also excluded.

All search results were imported into an Endnote 20 library. Duplicates were subsequently removed using the Yale Reference Deduplicator [10]. All studies were then imported into Covidence, a systematic review software, for screening. All titles and abstracts were independently screened by two coauthors (RM and LB). A full-text review for ultimate inclusion was conducted on all abstracts included by either reviewer. Disagreements were settled by a third author (JD).

Data Extraction and Quality Assessment

Data, including study populations, interventions, outcomes, quality, and follow-up period, were extracted from published articles into evidence tables by two coauthors (RM and LB) with oversight from additional coauthors (CG and JD). The primary and secondary outcomes for this review were pain/analgesic effect and safety, respectively. Study quality assessments were performed using two different quality assessment tools. The Newcastle-Ottawa Scale (NOS), which asks reviewers to score studies based on three elements (selection, comparability, and outcome) for a maximum score of nine points (lowest risk of bias), was used for included cohort studies [11]. The Cochrane risk-of-bias tool for randomized trials (RoB 2), which assesses the risk of bias in five different domains as well as assigns an overall risk of bias scoring (low risk, some concerns, or high risk of bias), was used to assess quality in included randomized trials [12,13]. Two authors individually assessed studies using the NOS (RM and LB) and RoB 2 (RM and CG) where appropriate. In both cases, authors completed assessments individually, and discrepancies between reviewers were resolved by consensus.

Analysis

Due to the heterogeneity of outcomes, pain and analgesic effects were assessed separately as cramp frequency, cramp severity, and non-cramp pain. Individual studies reported pain intensity using different scales, which were therefore analyzed using Hedge’s standardized mean difference (MD) [14]. In general, standardized MDs greater than 0.8 are considered to have large effects [15]. Pain frequency was reported as muscle spasms per week and was analyzed using the raw MD between treatments and controls. Due to the heterogeneity of treatment interventions, we did not pool study results but created forest plots showing individual study results. Statistical analyses were performed using Stata/BE, version 17.0 (StataCorp LLC, College Station, Texas, US).

Results

Study Selection

**Figure 1:**
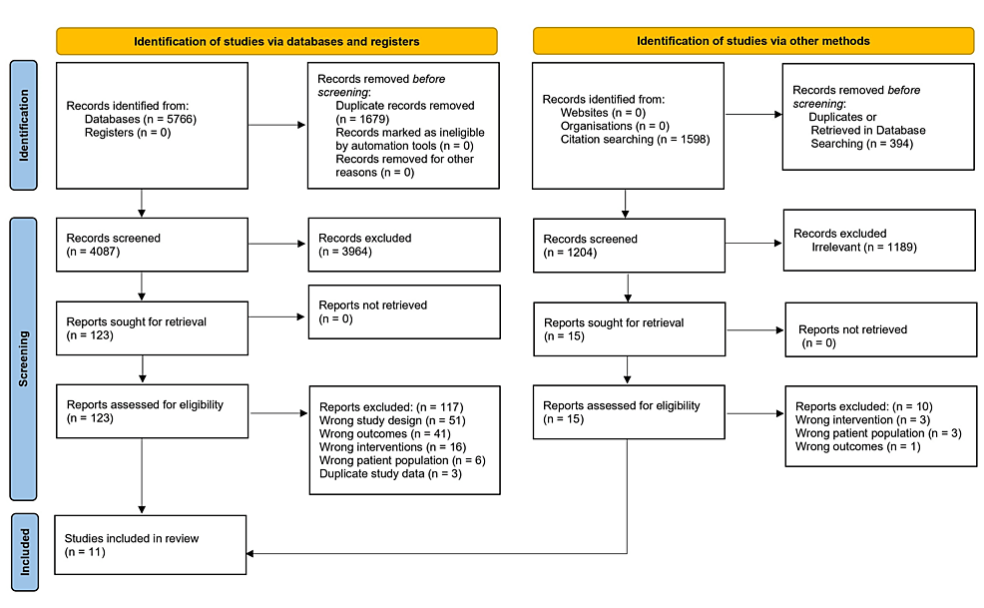
Evidence search and selection PRISMA, Preferred Reporting Items for Systematic Reviews and Meta-Analyse Adapted from Page et al. (2021) [27]

The electronic literature search identified 5,766 studies, of which 1,679 were duplicates. Of the remaining 4,087 studies, 123 were reviewed in full text, with 117 excluded for wrong study design, wrong outcomes, wrong interventions, wrong patient population, and duplicate study data (Figure 1). A table of excluded studies with reasons for exclusion can be downloaded from Open Science Framework (https://osf.io/57tpc). Five additional studies were identified through reference chasing, leaving a total of 11 included studies representing 340 participants [16-26].

Characteristics of Included Studies

Among the 11 included studies, six were single-arm trials [16-18,20,21,25], four were RCTs [22-24,26], and one used a prospective cohort design [19]. Geographically, the studies were varied, with three studies from the US [18,19,26], two studies from Japan [17,20], two studies from South Korea [21,25], one study from Brazil [24], one study from Australia [22], one study from Iran [23], and one study from Israel [16]. To define cirrhosis for participants, three studies used medical records of documented cirrhosis [18,19,21], three studies used clinical parameters [20,25,26], two used models for end-stage liver disease (MELD) scores [19,23], three used biopsy [16,17,20], four used laboratory testing parameters [18,20,23,26], two used Child-Pugh scores [16,24], one used laparoscopic examination [17], and three used imaging/radiology [17,20,26].

Interventions and exposures included oral taurine [22,25], oral vitamin E [16], niuche-shen-qi-wan [17], oral zinc sulfate [18], L-carnitine [20], electroacupuncture [21], curcumin [23], resistance training [24], and pickle juice [26]. Reference groups in controlled studies included placebo supplements [22,23], tap water [26], and no treatment [24]. One study was a prospective cohort study that examined pain and self-care behaviors, including pain medicine, tranquilizers, hot baths, reduced work schedules, napping, reduced activity, and more [19].

The studies included in our review demonstrated variability in pain assessments. Hansen et al. measured pain severity and pain interference using the Brief Pain Inventory [28], Nouri-Vaskeh et al. measured bodily pain using the 36-Item Short Form Survey (SF-36) [29], and Soldera et al. measured pain sensitivity using the SF-36 [24]. Eight studies included assessments of muscle cramps [16-18,20-22,25,26]. There was large variability in the measurement of the characteristics of cramps and the outcome measures used. Four studies measured the frequency of muscle cramps in days per week [17,18,20,22,25]. Seven studies measured muscle cramp severity and intensity. Two studies measured severity as mild, moderate, or severe [16,17]. Three studies measured severity on a 0-10 Visual Analogue Scale (VAS) [18,20,26]. One study measured cramp intensity on a 1-10 point Likert scale [22]. Three studies had a combined measurement based on the frequency and intensity of cramps. One study measured the proportion of cramp days with VAS less than five [26], and two used scoring systems based on frequency and intensity [16,25]. One study measured the duration of muscle cramps in minutes per week [22]. Finally, one study measured the time to cramp disappearance in days [17].

Safety

Mild adverse events were reported in four studies. One out of 12 participants reported epigastric discomfort but continued to take niushe-shen-qi-wan [17], one out of 12 participants reported mild watery stools while taking oral zinc sulfate [18], one of 10 participants experienced mild dyspepsia with oral taurine supplementation [25], and paracentesis was required in two participants (one in the intervention group and one in the control group) with a prior history of paracentesis before participating in the study [26] (Table 1).

**Table 1:** Characteristics of the included studies BPI, Brief Pain Inventory; ESLD, end-stage liver disease; MELD, model for end-stage liver disease; NR, not reported; RCT, randomized controlled trial; RET, resistance exercise training; SCB, Self-Care Behavior; SF-36, 36-item Short Form Health Survey; VAS, Visual Analogue Scale

Study and year	Study design	Patient population and database	Study participants	Follow-up period	Type of non-pharmacologic pain treatment	Pain assessment	Safety outcome
Konikoff et al. (1991) [16]	Single-arm trial	Patients with biopsy-proven cirrhosis in an outpatient liver clinic in Israel	29 patients (19 females). Mean age: 61.2 (without cramps) and 60.2 (with cramps)	4 weeks	Oral vitamin E (200 mg, 3x daily)	Severity of muscle cramps measured by a scoring system: Pain: mild 1, moderate 2, severe 3 points. Duration: seconds 1, minutes 2, hours 3 points. Frequency: 3 points, divided by the average time interval between cramps (in days)	Adverse effects
Motoo et al. (1997) [17]	Single-arm trial	Cirrhosis with muscle cramps. Kanazawa University, Japan	12 (4 decompensated, 8 compensated) patients. Pain site: legs for all 12. 7 females. Mean age: 65.1 (range: 50-73)	Evaluated at every 2-week check-up for at least 12 weeks	Niuche-shen-qi-wan (TJ-107) (herbal medicine) at a dose of 2.5 grams 3x a day for at least 2 weeks. All previous drugs were continued	Muscle cramp frequency (cramps per week) (only done at baseline) Severity: classified into 3 grades: severe (too painful to walk or move), moderate (mild restriction in daily life), and mild (no restriction in daily life). Time to cramp disappearance (days)	Side effects reported
Kugelmas (2000) [18]	Single-arm trial	Cirrhosis with muscle cramps at least twice weekly, hypozincemia, and awaiting liver transplant. University of Kentucky, Lexington	12 patients	Questionnaire given at baseline and after 12 weeks	Oral zinc sulfate. 220 mg twice daily for 12 weeks	Frequency of cramps (cramps per week). Pain/severity of cramp (scale 0-10)	Reported side effects
Hansen et al. (2014) [19]	Prospective cohort	Patients with ESLD and pain >3 and MELD >18. Oregon, USA	20 outpatients (15 males). Average age was 59 years (SD: 5.29, range 51-76)	Data collected at baseline and monthly over 6 months	Self-care behaviors: took pain medicine, asked for help, took tranquilizers, took a hot bath, reduced work hours, took a nap, reduced activity levels, etc.	BPI; SCB log	NR
Nakanishi et al. (2015) [20]	Single-arm trial	Patients with cirrhosis complicated by muscle cramps. 9/12-5/14. Tokyo, Japan	42 patients	Baseline and 8 weeks	300 mg of L-carnitine, 3 times/day (n = 19) or 4 times/day (n = 23), for 8 weeks	Frequency of muscle cramps (cramps per week). Severity of muscle cramp, measured by VAS	Adverse events
Seung-mo et al. (2018) [21]	Single-arm pilot trial	Patients with liver cirrhosis and muscle cramps. Daegu, Korea	14 outpatients (10 females). Mean age: 59.6 (SD: 6.4)	Baseline, visit 8, visit 14, and visit 15	Electroacupuncture 3 times a week for 4 weeks	Frequency of muscle cramps: Frequency category 1: “none, less than once a week, more than once a month, or less than once a month”; Frequency category 2: “more than once a day, more than once a week, less than once a day”	Adverse events
Vidot et al. (2018) [22]	Cross-over RCT	Cirrhosis with >3 or more muscle cramps per week. Australia	49 patients (30 completed the study). 21 males. Mean age: 54.7 (SD: 1.1)	Baseline, 4 weeks, and 8 weeks	Oral taurine: 500 mg twice daily for 2 weeks. 1,000 mg twice daily or placebo. Crossover to the alternative arm for 4 weeks	Frequency, duration, and intensity (1-10 Likert scale) of muscle cramps	Adverse events
Nouri-Vaskeh et al. (2020) [23]	Cross-over RCT	Cirrhosis patients MELD>11. Tabriz University, Iran	58 patients (30 females). Mean age: 46.0 (SD: 13.0) curcumin group and placebo group: 46.4 (SD: 10.6)	12 weeks	500 mg of curcumin twice daily for 12 weeks (n = 28) vs. placebo (n = 30)	Bodily pain (SF-36) and joint pain	Adverse effects
Soldera et al. (2020) [24]	Three-arm RCT	Patients with compensated cirrhosis. Caxias do Sul, Brazil	20 outpatients	Baseline, 6, 12, and 24 weeks	RET, low-intensity walking and stretching (ACG), and control (CG)	Percentage pain sensitivity improvement	Decompensation of cirrhosis
Jang et al. (2021) [25]	Single-arm trial	Cirrhosis with muscle cramps. Seoul, Korea	10 patients (6 males). Mean age: 66 (58-75)	Baseline, 2, 4, 6, and 8 weeks	Oral taurine 1 g/50 ml three times a day for 4 weeks	Frequency of muscle cramps (times/week) and muscle cramp score (frequency X intensity)	NR
Tapper et al. (2022) [26]	RCT	Patients with cirrhosis and a history of >4 muscle cramps in the previous month. University of Michigan, USA	74 patients. 58% male in the intervention group and 50% male in control. Mean age: 57.3 (SD: 12.5) intervention group and 55.8 (SD: 10.5) control	Cramps were assessed 10 times over 28 days using interactive text messages	Participants were randomized 1:1 to sips of pickle juice vs. tap water at cramp onset	Primary: change in cramp severity via VAS (0-10). Secondary: proportion of days with VAS cramps <5	Weight change, patient-reported paracentesis requirement

Cramp Frequency

**Figure 2:**
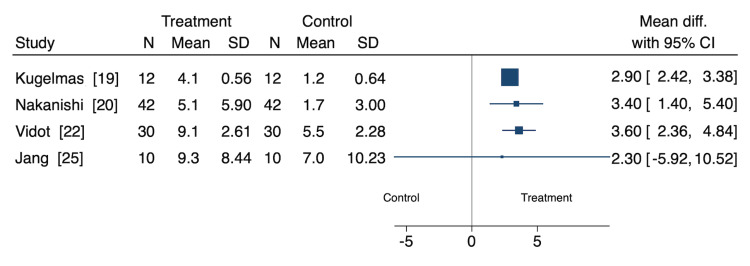
Cramp frequency

Four studies assessed the effect of nutritional and non-pharmacological interventions on cramp frequency (in cramps per week), representing 94 participants (Figure 2) [18,20,22,25]. Of these, three were cohort studies, and one was a RCT. Oral zinc sulfate (MD: 2.90 [95% CI: 2.42, 3.38]), L-carnitine (MD: 3.40 [95% CI: 1.40, 5.40]), and oral taurine (MD: 3.60 [95% CI: 2.36, 4.84]) interventions significantly decreased cramp cramps per week [18,20,22]. Jang et al. found oral taurine did not significantly affect cramps per week (MD: 2.30 [95% CI: -5.92, 10.52] [25].

Cramp Severity

**Figure 3:**
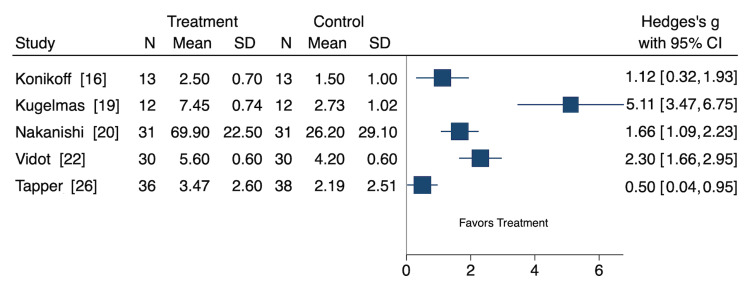
Cramp severity

Five studies assessed the effect of nutritional and non-pharmacological interventions on cramp severity, representing 122 participants (Figure 3) [16,18,20,22,26]. Of these, three were cohort studies, and two were RCTs. Because different studies used different severity scales, we used the standardized MD (SMD) to assess cramp severity. Oral vitamin E (SMD: 1.12 [CI: 0.32, 1.93]), oral zinc sulfate (SMD: 5.11 [CI: 3.47, 6.75]), L-carnitine (SMD: 1.66 [CI: 1.09, 2.23]), oral taurine (SMD: 2.30 [CI: 1.66, 2.95]), and pickle juice (SMD: 0.50 [CI: 0.04, 0.95]) interventions significantly decreased cramp severity [16,18,20,22,26].

Non-Cramp Pain

Two studies assessed non-cramp outcomes, with both studies being RCTs. In one study, curcumin supplementation was found to significantly decrease both bodily pain (p < 0.001) and joint pain (p = 0.017) compared to placebo [23]. In assessing pain sensitivity using the SF-36, resistance exercise training (baseline: 36%, week 24: 74%) and an active control group (baseline: 63%, week 24: 76%) both improved compared to the control group (baseline: 57%, week 24: 52%) [24]. Differences between groups were not assessed for statistical significance (Table 2).

**Table 2:** Study outcomes ACG, active control group (low intensity walking and stretching); BPI, Brief Pain Inventory; CG, control group; NR, not reported; RET, resistance exercise training group; VAS, Visual Analogue Scale

Study and year	Pain outcome #1	Pain outcome #2	Safety
Konikoff et al. (1991) [16]	Cramp score: Baseline: 5.2 (0.8) Post: 3.1 (1.9) p-value: 0.005 Patients with subnormal (n = 5) vitamin E levels at baseline improved the most from treatment (cramp score 5.1 at baseline to 1.8 post)	Severity: Baseline: 2.5 (0.7) Post: 1.5 (1.0) p-value: 0.002. Frequency: Baseline: 0.8 (0.5) Post: 0.3 (0.4) p-value: 0.01. Duration: Baseline: 2.0 (0.2) Post: 1.3 (0.8) p-value: 0.04	No adverse effects were reported by patients
Motoo et al. (1997) [17]	Cramp disappearance occurred in 12/12 participants. Average period of disappearance of cramps: 10.5 days	N/A	1/12 participants experienced epigastric discomfort but continued to take TJ-107
Kugelmas (2000) [18]	Cramp frequency (times per week): Baseline: 4.09 (0.56) Post: 1.18 (0.64)	Cramp severity (0-10): Baseline: 7.45 (0.74) Post: 2.73 (1.02)	Mild watery diarrhea (n = 1). No reports of nausea
Hansen et al. (2014) [19]	Severity of pain (BPI): means ranged from 5.36 to 6.64 (1.89-2.32)	Pain interference (BPI): 5.36 to 6.64 (2.58-3.11)	NR
Nakanishi et al. (2015) [20]	Overall frequency of muscle cramps per week from baseline to 8 weeks reduced from 5.1 +/- 5.9 to 1.7 +/- 3.0 (p = 0.0019). Cramp reduction occurred in 88.1% of patients. Cramps disappeared in 28.6% of patients. 900 mg vs. 1,200 mg: The rate of disappearance of muscle cramps was significantly higher in the 1,200 mg group (43.5% vs. 10.5%) (p = 0.037)	Overall mean VAS score. Baseline: 69.9 +/- 22.5 8 weeks: 26.2 +/- 29.1 p < 0.0001 900 mg vs. 1200 mg VAS scores were significantly lower in 1,200 mg group after 8 weeks (9.9 +/- 13.5 vs. 39.6 +/- 38.1) (p = 0.003)	No adverse events were reported
Seung-mo et al. (2018) [21]	Frequency category 1 - N (%) Baseline: 0 (0); visit 8: 6 (42.9); visit 14: 10 (71.4); visit 15: 12 (85.7). Frequency category 2 – N (%) Baseline: 14 (100); visit 8: 8 (57.1); visit 14: 4 (28.6); visit 15: 2 (14.3). Effectively reduced muscle cramps: p = 0.000”=	N/A	No adverse events reported
Vidot et al. (2018) [22]	Frequency of cramps (number per week) Baseline: 13.2 (1.7) Period 1 placebo: 8.6 (2.0)* Period 1 taurine: 6.8 (2.0)** Period 2 placebo: 9.6 (3.1)** Period 2 taurine: 4.2 (1.1)*. Cramp duration (minutes per week) Baseline: 86.5 (14.7) Period 1 placebo: 44.4 (10.9) Period 1 taurine: 57.9 (19.9) Period 2 placebo: 120.4 (39.6) Period 2 taurine: 21.1 (7.0)*,** *: p < 0.05 between placebo and treatment **: p < 0.05 between baseline and treatment	Intensity of cramps (1-10 Likert scale) Baseline: 6.5 (0.5) Period 1 placebo: 5.8 (0.6) Period 1 taurine: 4.1 (0.6)* ,** Period 2 placebo: 5.4 (0.6) Period 2 taurine: 4.3 (0.6)**. *: p < 0.05 between placebo and treatment; **: p < 0.05 between baseline and treatment	No adverse side effects associated with taurine supplementation
Nouri-Vaskeh et al. (2020) [23]	Bodily Pain Curcumin group Baseline: 48.93 (12.06) 12 weeks: 57.57 (12.64). Placebo group Baseline: 49.50 (10.27) 12 weeks: 47.33 (9.69) p < 0.001 (improved)	Joint Pain Curcumin group Baseline: 0.42 (0.64) 12 weeks: 0.28 (0.55). Placebo group Baseline: 0.26 (0.43) 12 weeks: 0.46 (0.64) p = 0.017	No adverse effects declared
Soldera et al. (2020) [24]	Pain sensitivity (SF-36). Baseline: RET 36%; ACG 63%; CG 57%; Week 24: RET 74%; ACG 76%; CG 52%	N/A	No decompensation of cirrhosis
Jang et al. (2021) [25]	Muscle cramp frequency (times/week): Mean (SD) Baseline: 9.3 (8.4) Week 4: 6.3 (8.3) Week 8: 7 (9.7)	Muscle cramp score (frequency multiplied by intensity) Baseline: 43.2 (40.7) Week 4: 18.6 (32.0) Week 8: 24.45 (33.7)	1 participant experienced mild dyspepsia associated with the study drug
Tapper et al. (2022) [26]	Change in cramp severity (VAS) Pickle juice: -2.25 (3.61) Control: -0.36 (2.87) p-value: 0.03	The proportion of cramp days with VAS <5: 46% pickle juice vs. 35% control (p = 0.2)	No patient required a first paracentesis in the study period. Among those with prior paracentesis, one required paracentesis during the study period in each arm (n = 2)

Quality Appraisal

Overall, cohort studies were of similar quality (NOS score range: 3-4). Of the seven cohort studies, six scored four points, and one study scored three points [16-21,25]. The main reasons cohort studies had a decrease in points were for how the study population was selected and/or for lacking a control group. Of the four randomized trials, two had some concerns and two had a high risk of bias [22-24,26]. The main domain receiving scores of “some concerns” was missing outcome data, while the domain receiving the most scores of “high risk of bias” was the measurement of outcome due to a lack of blinding of study participants (Table 3).

**Table 3:** Study quality assessment H, high risk of bias; L, low risk of bias; NOS, Newcastle-Ottawa Scale; RCT, randomized controlled trial; RoB, risk of bias; S, some concerns

Author and year	Selection (4)	Comparability (2)	Outcome (3)	NOS score	Comment		
Cohort studies – NOS [11]							
Konikoff et al. (1991) [16]	2	0	2	4/9	Vitamin E; muscle cramps		
Motoo et al. (1997) [17]	2	0	2	4/9	Niuche-shen-qi-wan; muscle cramps		
Kugelmas (2000) [18]	2	0	2	4/9	Oral zinc sulfate; muscle cramps		
Hansen et al. (2014) [19]	2	0	1	3/9	Pain and self-care behaviors (longitudinal)		
Nakanishi et al. (2015) [20]	2	0	2	4/9	L-carnitine; muscle cramps		
Seung-mo et al. (2018) [21]	2	0	2	4/9	Electroacupuncture; muscle cramps		
Jang et al. (2021) [25]	2	0	2	4/9	Oral taurine; muscle cramps		
RCTs – Cochrane RoB2 tool [12]							
Author and year	Randomization process	Deviations from interventions	Missing outcome data	Measurement of outcome	Selective reporting	Overall RoB	Comment
Vidot et al. (2018) [22]	L	L	S	L	S	S	Oral taurine; muscle cramps
Nouri-Vaskeh et al. (2020) [23]	L	L	S	L	S	S	Curcumin; bodily and joint pain
Soldera et al. (2020) [24]	S	L	S	H	L	H	Resistance training; pain sensitivity
Tapper et al. (2022) [26]	L	L	L	H	L	H	Pickle juice; muscle cramps

Discussion

Key Findings

This systematic review found that limited information is available in the published literature assessing nutritional and non-pharmacological interventions for the treatment of pain in patients with cirrhosis. We included 11 studies, with the majority being cohort designs, assessing the impact non-pharmacological and nutritional interventions have on the frequency (four studies) and severity (five studies) of muscle cramps in patients with cirrhosis, and two studies assessing non-cramp pain. Multiple interventions were found to improve muscle cramp frequency and severity, with some mixed results. The risk of bias was moderate to high for all included studies, largely due to study design, missing data, and a lack of blinding of study participants.

Interpretation

Oral taurine [22,25], vitamin E [16], oral zinc sulfate [18], L-carnitine [20], and pickle juice [26] decreased cramp severity. Cramp frequency decreased with vitamin E [16], oral zinc sulfate [18], L-carnitine [20], niushe-shen-qi-wan [17], electroacupuncture [21], and oral taurine [22,25] interventions, while the duration of cramps decreased with vitamin E [16] and oral taurine administration [22]. However, some interventions showed mixed effects. One study by Jang et al. [25] found treatment with oral taurine did not have a significant effect on cramp frequency, while another by Vidot et al. found taurine to significantly decrease cramp frequency [22]. Furthermore, Tapper et al. [26] found a pickle juice intervention to decrease cramp severity (assessed via VAS) but found the difference between intervention and control groups for the proportion of cramp days with VAS less than five to be statistically non-significant.

Only two included studies evaluated the use of nutritional and non-pharmacological interventions to address non-cramp pain. Curcumin was found to improve bodily and joint pain [23]. Additionally, resistance training and low-intensity stretching and walking programs improved pain sensitivity [24]. More research is needed in this area, as previous work has found the prevalence of pain in patients with end-stage liver disease to be as high as 79% and the prevalence of muscle cramps to be as high as 68% [30].

Our study also found only minor adverse events associated with interventions. Adverse events were mild, rare, and consisted of reports of epigastric discomfort and dyspepsia. These results suggest that the use of nutritional and non-pharmacological interventions for pain and cramping in patients with cirrhosis is safe. However, several different treatments were included in this review, and sample sizes were often small. More studies, with larger sample sizes, are needed to better understand the safety of nutritional and non-pharmacological interventions in this population.

There was significant heterogeneity in the interventions used in the included studies. Only two of the 11 studies used the same intervention (oral taurine) [22,25]. Many interventional studies had small sample sizes and often did not include a control or comparison group. More studies should be conducted using each intervention with larger sample sizes and the inclusion of control groups to gain a better understanding of the efficacy of nutritional and non-pharmacological interventions. Furthermore, we found a lack of standardization of outcome measures and pain characteristics assessed across studies. Future work could benefit from the inclusion of both cramp and non-cramp pain assessments, as well as the validation of pain-related outcome measures in this population.

Limitations and Future Research Directions

The limitations of this work are that we only included published studies and studies written in English and therefore may have excluded some results. Furthermore, many participants in these studies had medical comorbidities that may have affected the results. The risk of bias was moderate to high for the included studies and may have impacted the results. Selection of study populations, studies lacking control groups, and difficulties with blinding (e.g., pickle juice intervention) were all sources of bias that warranted careful interpretation of study findings. Finally, nutritional and non-pharmacological interventions were short in duration, leaving unanswered questions about the efficacy and safety of long-term use.

Continued research in the realm of nutritional and non-pharmacological interventions for pain management in patients with cirrhosis can benefit from standardization in treatment delivery and definitions of pain outcomes. Large-scale longitudinal studies evaluating the association of nutritional and non-pharmacological interventions for pain management are needed, and examination of potential subgroups at higher risk for safety concerns based on severity of liver disease and/or comorbidities is warranted. Many patients request nutritional and non-pharmacological treatments for chronic pain conditions, and more research is needed in this area.

## Conclusions

This systematic review found that the frequency and severity of muscle cramps were more frequently investigated than non-cramp pain in patients with cirrhosis. The findings suggest that nutritional and non-pharmacological interventions may be safe and effective for the treatment of pain and painful muscle cramps in patients with cirrhosis. However, studies often did not contain control or comparator interventions, and only two studies examined the same type of treatment. Further research is needed to determine the efficacy, safety, and optimal frequency and dosage of nutritional and non-pharmacological interventions for pain and painful muscle cramps in patients with cirrhosis.

## Appendices

Appendix A

**Table 4:** PRISMA 2020 main checklist Adapted from Page et al. (2021) [27]

Topic	No.	Item	Location where item is reported
TITLE			
Title	1	Identify the report as a systematic review.	LN1-2
ABSTRACT			
Abstract	2	See the PRISMA 2020 for Abstracts checklist	LN26-44
INTRODUCTION			
Rationale	3	Describe the rationale for the review in the context of existing knowledge.	LN46-59
Objectives	4	Provide an explicit statement of the objective(s) or question(s) the review addresses.	LN59-61
METHODS			
Eligibility criteria	5	Specify the inclusion and exclusion criteria for the review and how studies were grouped for the syntheses.	LN82-92
Information sources	6	Specify all databases, registers, websites, organisations, reference lists and other sources searched or consulted to identify studies. Specify the date when each source was last searched or consulted.	LN70-79
Search strategy	7	Present the full search strategies for all databases, registers and websites, including any filters and limits used.	Suppl. 2
Selection process	8	Specify the methods used to decide whether a study met the inclusion criteria of the review, including how many reviewers screened each record and each report retrieved, whether they worked independently, and if applicable, details of automation tools used in the process.	LN94-99
Data collection process	9	Specify the methods used to collect data from reports, including how many reviewers collected data from each report, whether they worked independently, any processes for obtaining or confirming data from study investigators, and if applicable, details of automation tools used in the process.	LN102-103
Data items	10a	List and define all outcomes for which data were sought. Specify whether all results that were compatible with each outcome domain in each study were sought (e.g. for all measures, time points, analyses), and if not, the methods used to decide which results to collect.	LN103-104
	10b	List and define all other variables for which data were sought (e.g. participant and intervention characteristics, funding sources). Describe any assumptions made about any missing or unclear information.	LN103-104
Study risk of bias assessment	11	Specify the methods used to assess risk of bias in the included studies, including details of the tool(s) used, how many reviewers assessed each study and whether they worked independently, and if applicable, details of automation tools used in the process.	Table 3
Effect measures	12	Specify for each outcome the effect measure(s) (e.g. risk ratio, mean difference) used in the synthesis or presentation of results.	LN116-122
Synthesis methods	13a	Describe the processes used to decide which studies were eligible for each synthesis (e.g. tabulating the study intervention characteristics and comparing against the planned groups for each synthesis (item 5)).	LN102-104, 116-122
	13b	Describe any methods required to prepare the data for presentation or synthesis, such as handling of missing summary statistics, or data conversions.	LN116-122
	13c	Describe any methods used to tabulate or visually display results of individual studies and syntheses.	LN116-122
	13d	Describe any methods used to synthesize results and provide a rationale for the choice(s). If meta-analysis was performed, describe the model(s), method(s) to identify the presence and extent of statistical heterogeneity, and software package(s) used.	LN116-122
	13e	Describe any methods used to explore possible causes of heterogeneity among study results (e.g. subgroup analysis, meta-regression).	LN116-122
	13f	Describe any sensitivity analyses conducted to assess robustness of the synthesized results.	LN116-122
Reporting bias assessment	14	Describe any methods used to assess risk of bias due to missing results in a synthesis (arising from reporting biases).	LN108-113
Certainty assessment	15	Describe any methods used to assess certainty (or confidence) in the body of evidence for an outcome.	LN116-122
RESULTS			
Study selection	16a	Describe the results of the search and selection process, from the number of records identified in the search to the number of studies included in the review, ideally using a flow diagram.	LN126-131
	16b	Cite studies that might appear to meet the inclusion criteria, but which were excluded, and explain why they were excluded.	Suppl. 3
Study characteristics	17	Cite each included study and present its characteristics.	LN133-160
Risk of bias in studies	18	Present assessments of risk of bias for each included study.	LN196-202
Results of individual studies	19	For all outcomes, present, for each study: (a) summary statistics for each group (where appropriate) and (b) an effect estimate and its precision (e.g. confidence/credible interval), ideally using structured tables or plots.	Table 2
Results of syntheses	20a	For each synthesis, briefly summarise the characteristics and risk of bias among contributing studies.	LN163-202
	20b	Present results of all statistical syntheses conducted. If meta-analysis was done, present for each the summary estimate and its precision (e.g. confidence/credible interval) and measures of statistical heterogeneity. If comparing groups, describe the direction of the effect.	LN163-202
	20c	Present results of all investigations of possible causes of heterogeneity among study results.	LN163-202
	20d	Present results of all sensitivity analyses conducted to assess the robustness of the synthesized results.	LN163-202
Reporting biases	21	Present assessments of risk of bias due to missing results (arising from reporting biases) for each synthesis assessed.	LN196-202
Certainty of evidence	22	Present assessments of certainty (or confidence) in the body of evidence for each outcome assessed.	LN196-202
DISCUSSION			
Discussion	23a	Provide a general interpretation of the results in the context of other evidence.	LN205-244
	23b	Discuss any limitations of the evidence included in the review.	LN248-256
	23c	Discuss any limitations of the review processes used.	LN246-248
	23d	Discuss implications of the results for practice, policy, and future research.	LN259-266
OTHER INFORMATION			
Registration and protocol	24a	Provide registration information for the review, including register name and registration number, or state that the review was not registered.	LN66-67
	24b	Indicate where the review protocol can be accessed, or state that a protocol was not prepared.	LN66-67
	24c	Describe and explain any amendments to information provided at registration or in the protocol.	LN66-67
Support	25	Describe sources of financial or non-financial support for the review, and the role of the funders or sponsors in the review.	LN268-273
Competing interests	26	Declare any competing interests of review authors.	LN268-273
Availability of data, code and other materials	27	Report which of the following are publicly available and where they can be found: template data collection forms; data extracted from included studies; data used for all analyses; analytic code; any other materials used in the review.	Upon request

Appendix B

**Table 5:** PRISMA abstract checklist Adapted from Page et al. (2021) [27]

Topic	No.	Item	Reported?
TITLE			
Title	1	Identify the report as a systematic review.	Yes
BACKGROUND			
Objectives	2	Provide an explicit statement of the main objective(s) or question(s) the review addresses.	Yes
METHODS			
Eligibility criteria	3	Specify the inclusion and exclusion criteria for the review.	Yes
Information sources	4	Specify the information sources (e.g. databases, registers) used to identify studies and the date when each was last searched.	Yes
Risk of bias	5	Specify the methods used to assess risk of bias in the included studies.	Yes
Synthesis of results	6	Specify the methods used to present and synthesize results.	Yes
RESULTS			
Included studies	7	Give the total number of included studies and participants and summarise relevant characteristics of studies.	Yes
Synthesis of results	8	Present results for main outcomes, preferably indicating the number of included studies and participants for each. If meta-analysis was done, report the summary estimate and confidence/credible interval. If comparing groups, indicate the direction of the effect (i.e. which group is favoured).	Yes
DISCUSSION			
Limitations of evidence	9	Provide a brief summary of the limitations of the evidence included in the review (e.g. study risk of bias, inconsistency and imprecision).	Yes
Interpretation	10	Provide a general interpretation of the results and important implications.	Yes
OTHER			
Funding	11	Specify the primary source of funding for the review.	Yes
Registration	12	Provide the register name and registration number.	Yes

Appendix C

**Table 6:** MOOSE checklist for meta-analyses of observational studies Adapted from Stroup et al. (2000) [5]

Item No.	Recommendation	Reported on Page No
Reporting of background should include		
1	Problem definition	3
2	Hypothesis statement	3
3	Description of study outcome(s)	3
4	Type of exposure or intervention used	3
5	Type of study designs used	3
6	Study population	3
Reporting of search strategy should include		
7	Qualifications of searchers (eg, librarians and investigators)	4
8	Search strategy, including time period included in the synthesis and key words	4, Suppl. 2
9	Effort to include all available studies, including contact with authors	N/A
10	Databases and registries searched	4
11	Search software used, name and version, including special features used (eg, explosion)	4
12	Use of hand searching (eg, reference lists of obtained articles)	4
13	List of citations located and those excluded, including justification	4
14	Method of addressing articles published in languages other than English	4
15	Method of handling abstracts and unpublished studies	4
16	Description of any contact with authors	N/A
Reporting of methods should include		
17	Description of relevance or appropriateness of studies assembled for assessing the hypothesis to be tested	4-5
18	Rationale for the selection and coding of data (eg, sound clinical principles or convenience)	4-5
19	Documentation of how data were classified and coded (eg, multiple raters, blinding and interrater reliability)	4-5
20	Assessment of confounding (eg, comparability of cases and controls in studies where appropriate)	4-5
21	Assessment of study quality, including blinding of quality assessors, stratification or regression on possible predictors of study results	5
22	Assessment of heterogeneity	5
23	Description of statistical methods (eg, complete description of fixed or random effects models, justification of whether the chosen models account for predictors of study results, dose-response models, or cumulative meta-analysis) in sufficient detail to be replicated	6
24	Provision of appropriate tables and graphics	6
Reporting of results should include		
25	Graphic summarizing individual study estimates and overall estimate	Figure 1
26	Table giving descriptive information for each study included	Table 1
27	Results of sensitivity testing (eg, subgroup analysis)	Figure 2-3
28	Indication of statistical uncertainty of findings	6
Reporting of discussion should include		
29	Quantitative assessment of bias (eg, publication bias)	10
30	Justification for exclusion (eg, exclusion of non-English language citations)	11
31	Assessment of quality of included studies	11
Reporting of conclusions should include		
32	Consideration of alternative explanations for observed results	11-12
33	Generalization of the conclusions (ie, appropriate for the data presented and within the domain of the literature review)	12
34	Guidelines for future research	12
35	Disclosure of funding source	12-13
